# The midwife–woman relationship in a South Wales community: Experiences of midwives and migrant Pakistani women in early pregnancy

**DOI:** 10.1111/hex.12629

**Published:** 2017-09-29

**Authors:** Laura Goodwin, Billie Hunter, Aled Jones

**Affiliations:** ^1^ Institute of Applied Health Research University of Birmingham Birmingham UK; ^2^ School of Healthcare Sciences Cardiff University Cardiff UK

**Keywords:** health‐care relationships, immigration, inequality, maternity, midwifery, migrant women, pregnancy

## Abstract

**Background:**

In 2015, 27.5% of births in England and Wales were to mothers born outside of the UK. Compared to their White British peers, minority ethnic and migrant women are at a significantly higher risk of maternal and perinatal mortality, along with lower maternity care satisfaction. Existing literature highlights the importance of midwife–woman relationships in care satisfaction and pregnancy outcomes; however, little research has explored midwife–woman relationships for migrant and minority ethnic women in the UK.

**Methods:**

A focused ethnography was conducted in South Wales, UK, including semi‐structured interviews with 9 migrant Pakistani participants and 11 practising midwives, fieldwork in the local migrant Pakistani community and local maternity services, observations of antenatal appointments, and reviews of relevant media. Thematic data analysis was undertaken concurrently with data collection.

**Findings:**

The midwife–woman relationship was important for participants' experiences of care. Numerous social and ecological factors influenced this relationship, including family relationships, culture and religion, differing health‐care systems, authoritative knowledge and communication of information. Marked differences were seen between midwives and women in the perceived importance of these factors.

**Conclusions:**

Findings provide new theoretical insights into the complex factors contributing to the health‐care expectations of pregnant migrant Pakistani women in the UK. These findings may be used to create meaningful dialogue between women and midwives, encourage women's involvement in decisions about their health care and facilitate future midwifery education and research. Conclusions are relevant to a broad international audience, as achieving better outcomes for migrant and ethnic minority communities is of global concern.

## 1 BACKGROUND

Non‐UK‐born communities continue to grow within the United Kingdom.^1^ Indeed, a recent report from the migration observatory suggests that one in seven (13.1%) of the UK population in 2014 were born abroad,^2^ and in 2015, over a quarter of births (27.5%) in England and Wales were to mothers born outside of the UK.^3^ Furthermore, during 2015, the number of births to non‐UK‐born women in England and Wales increased by 2.5% from the previous year, whilst births to UK‐born women decreased by 0.4%.^3^

In the UK, minority ethnic and migrant women consistently report lower maternity care satisfaction^4^, ^5^, ^6^, ^7^ and less choice in their maternity care^4^, ^8^ than their White British counterparts. In addition to poor experiences of maternity care in the UK, a wealth of research details poor pregnancy outcomes for these women, including an increased risk of complications during pregnancy,^9^ unplanned caesarean section^10^ and having their baby cared for in a neonatal unit.^11^ Substantially higher maternal mortality rates are also observed for minority ethnic and migrant women.^12^ For example, between 2011 and 2013, the estimated mortality rate for White women in England was 7.8 deaths per 100 000 maternities;^12^ for Black women, this rate was more than tripled at 28.3^12^ and was also significantly higher for both Pakistani and Bangladeshi women, 15.9 and 14.7, respectively.^12^ Furthermore, the risk of maternal mortality seems to be increasing for some migrant women in the UK: between 2011 and 2014, the relative risk of maternal mortality for Pakistan‐born women living in the UK increased from 1.53 to 2.24.^12^, ^13^ Data consistently suggest that minority ethnic and migrant women are also at an increased risk of perinatal mortality. In 2013, mothers of Black ethnic origin were twice as likely to have a stillbirth than mothers of White ethnic origin,^14^ and women of Asian or Asian British ethnic origin had up to 64% higher stillbirth rates than their White counterparts.^15^

Previous research has attempted to ascertain why these differences exist, and suggests that risk factors for poor outcomes such as access to health care, racism, cultural beliefs and poor underlying health are likely to impact migrant women to a higher degree than UK‐born minority ethnic women.^16^ For example, Hayes et al^16^ suggest that women who have recently arrived in the host country and who do not know how to, or cannot, legitimately access care are those most at risk of negative maternity outcomes. This view is echoed by women^17^ and health‐care professionals^18^ alike; UK‐born minority ethnic women felt that being born in the UK allowed them a better understanding of how to access care and information,^18^ and maternity care professionals suggested that women's language competency and familiarity with the system were “key advantages” in care provision.^17^ Poor social networks, commonly seen in new migrant populations,^16^ have also been proposed as a risk for substandard maternity outcomes.^16^, ^19^ Associations have also been suggested between poor pregnancy outcomes and factors such as genetic risk,^20^, ^21^ differences in socioeconomic status,^22^ language barriers^23^ and stereotyping/racism.^24^ However, ethnic inequality in these outcomes remains even once these contributing factors have been accounted for.^25^, ^26^ Consequently, it would seem that additional, largely unexplored, explanations may exist for the observed differences in pregnancy outcomes.^26^

Existing literature suggests an association between midwife–woman relationships and pregnancy outcomes,^27^ impacting not only on uptake of antenatal care,^27^, ^28^ but also influencing the quality of care received once services have been accessed.^17^, ^29^, ^30^, ^31^ There is a strong suggestion in the literature that a poor relationship may result in poor outcomes for women.^29^ Despite this, research exploring midwife–woman relationships is limited. This is surprising considering the importance placed on the quality of the relationship by both women and midwives in previous research.^26^ The limited research suggests that midwives may have more difficult relationships with migrant and minority ethnic women, compared to their White British counterparts.^32^, ^33^ It is possible, therefore, that poorer quality of midwife–woman relationships for minority ethnic and migrant women may present an alternative contributing factor towards ethnic inequalities in outcomes, a perspective that has largely been under‐explored in previous studies.^26^

As such, this study was designed to address the paucity of literature examining midwife–woman relationships for migrant women by exploring relationships between first‐generation migrant women and midwives in the South Wales region of the UK, focusing on identifying the factors contributing to these relationships, and the ways in which these relationships might affect women's experiences of care. The focus was on Pakistani women specifically, as mortality reports published at the time of study design suggested that Pakistani women were at significantly increased risk of both infant mortality^34^ and maternal mortality^29^ when compared to all other ethnic groups in the UK.

## 2 METHODS

**Figure 1:**
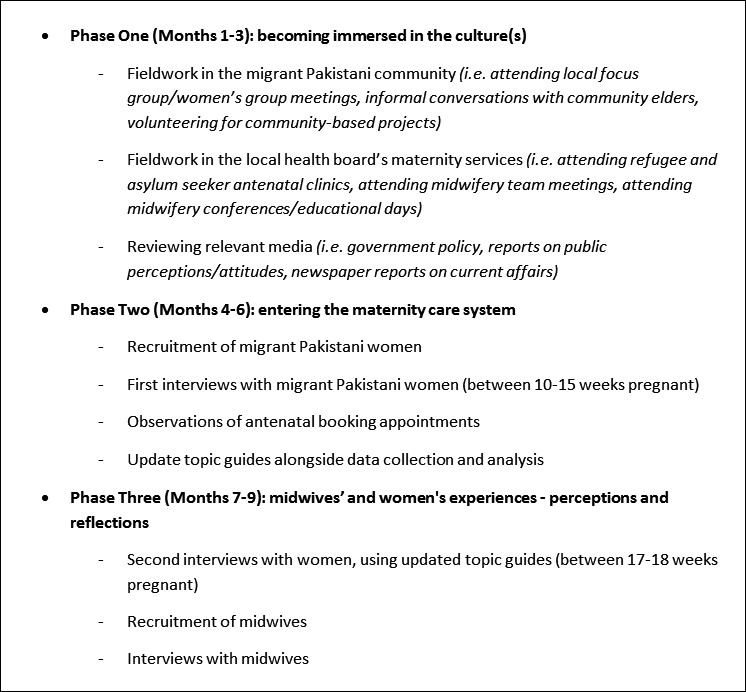
Data collection schedule

Given the exploratory nature of the enquiry and the limited existing evidence base, a focused ethnographic approach, using qualitative research methods, was taken to data collection (a full data collection schedule is presented in Figure 1). The ethnographic approach is of particular relevance to the current research question, as it was developed as a way to understand the social life of humans within specific cultures^35^ and analyse cultural norms,^36^ allowing for cross‐cultural comparison and providing a better understanding of behavioural differences and intergroup conflicts.^35^, ^37^ Focused ethnography differs to the traditional ethnographic approach, in that it is conducted within a discrete community or context, whereby participants have specific knowledge about an identified phenomenon.^38^ In this way, focused ethnography aims to explore participants' beliefs and practices by viewing them within the context in which they actually occur.^39^ As such, proponents of focussed ethnography argue that it is especially well suited to studying the practice of health care as a cultural phenomenon and to understanding the meaning that members of a subculture or group assign to their experiences.^40^ The focused ethnographic approach has, therefore, been increasingly adopted in health services research,^41^ as it can assist health‐care practitioners to identify and meet the needs of individuals from a certain culture by giving insight to behavioural differences.^42^

In line with the ethnographic approach and to enhance thickness of data,^39^, ^41^, ^43^ several data collection methods were utilized (Figure 1). These included the following: (i) preliminary fieldwork in the communities under study; (ii) reviews of relevant media (ie newspaper articles, policies); (iii) semi‐structured interviews; (iv) non‐participant observations of antenatal booking appointments; (v) reflexive fieldnotes (written throughout study design, recruitment data collection and analysis). Preliminary fieldwork consisted of approximately 80 hours of participation in activities and events in migrant Pakistani communities and local maternity services over a 3‐month period, which were designed to facilitate embeddedness in the cultural worlds of both participant groups (migrant Pakistani women and UK midwives), in order to “interpret the world in the way they do” [Ref.^35^, p.8]. As recommended by other health service researchers,^32^, ^41^ more formal observation periods would be undertaken at a later stage of the study, alongside in‐depth interviews, to facilitate strategic data collection as the research questions became progressively more focused. Interviews and observations were repeated to capture any changes in midwife–woman relationships over time,^44^ and reflexive accounts were written and shared with a project support group (described below) to ensure that all potential personal and interpersonal influences were explored and considered appropriately.

Ethical approval was granted by the NHS Research Ethics Committee (REC) for Wales, and the research protocol was reviewed by the affiliated University, and the study site. A project support group was set up to advise on all aspects of the study methods (eg design, recruitment and data analysis). This included a Consultant Midwife from the local health board, a member of Race Equality First, and the first author's research supervisors (authors 2 and 3).

### 2.1 Participants and recruitment procedures

The study site was a maternity unit in South Wales, which provides care to around 6000 women annually. Services include an alongside midwifery‐led unit, and tertiary foetal medicine and neonatal services. The health board employs around 14 000 staff in total, including around 300 midwives.^45^ In the city served by this health board, 80% of the population report their ethnicity as “White British.” The other main ethnic groups are “Other White” (4%), “Indian” (2%), “Pakistani” (2%) and “African” (2%).^46^

For interviews, participants included 7 first‐generation migrant Pakistani women receiving maternity care in South Wales and 11 midwives with experience of providing maternity care to migrant Pakistani women. Inclusion/exclusion criteria are outlined in Table 1. Naturally occurring interviews resulted in data also being included from the mother of one of the participants (n = 1) and a migrant Pakistani interpreter (n = 1). The number of interviews was not predefined but was limited to an extent by the planned duration for data collection; however, no new concepts were emerging from the data when recruitment stopped.^47^ For observational periods in antenatal clinics (totalling approximately 14 hours), participants included 7 midwives (2 of whom participated in interviews) and 15 women (2 migrant Pakistani women who participated in interviews, 4 migrant Pakistani women who were not interviewed and 9 UK‐born women of varying ethnicity).

**Table 1:** Inclusion/exclusion criteria for participants

Participant group	Inclusion criteria	Exclusion criteria
Midwives	UK‐born Working in South Wales Experience of providing maternity care to migrant Pakistani women	
Migrant Pakistani women	Born in Pakistan Between 3‐6 mo pregnant at the time of recruitment Receiving maternity care in South Wales for the first time Aged 16‐45	Serious illness/conditions which may affect the pregnancy

A purposive sampling approach^48^ was taken to recruiting both women and midwives. All eligible migrant Pakistani women were identified by midwives via antenatal clinic booking records, and midwives provided these women with participant information packs at their next appointment. Packs included written participant information in both English and Urdu, along with a CD containing audio tracks and YouTube videos of participant information in Pashto, Punjabi and Urdu. Migrant Pakistani women were interviewed at two time‐points, once after their first antenatal appointment, and then again after their second/third antenatal appointment. Interviews lasted between 20 and 90 minutes, and a flexible topic guide was used to guide the conversation. The aim of the first set of interviews was to explore women's initial expectations of maternity care in South Wales, and whether these expectations were met during their first contact with their midwives. The second set of interviews explored how women's initial expectations of maternity care in South Wales were managed throughout their pregnancy, and how women perceived their midwife–woman relationship to have affected the pregnancy so far. Language interpreters were offered to all women at all points of engagement with the study. In cases where an interpreter was used (n = 2), the researcher would ask a question, which the interpreter would translate into the required language (in both cases this was Urdu). The woman would then reply in Urdu, which the interpreter would translate back to English for the researcher. Anonymized audio extracts of interviews were sent to an independent interpreter for validation.

To recruit midwives, invitations to participate were initially sent out by the Head of Midwifery, with a request to contact the researcher to express interest. A snowballing approach was then used to recruit other midwives eligible for participation. Interviews with midwives lasted between 20 and 60 minutes and explored their experiences of working with migrant clients and with Pakistani women specifically. Midwives were asked about their relationships with these women and were asked to discuss the barriers and facilitators to establishing these relationships.

A total of 15 observations of antenatal booking appointments (20‐60 minutes each) took place in antenatal clinics across the local health board over a period of 3‐6 months. In line with the focused ethnographic approach, the enquiry became progressively focused on specific research questions as data collection progressed.^41^ In practice, this meant that flexible “observation guides” (prompts about what to focus observations on) were gradually developed to collect data more purposively.^38^ These guides were informed by initial observation data, interview data and previous observation research in maternity care.^49^

### 2.2 Analysis

Thematic analysis^50^ of the data was undertaken, resulting in a data‐driven inductive approach. The first step of the analysis was to personally transcribe and listen to interviews in their entirety, at least 3‐4 times. Next, early themes and topics of importance to participants were highlighted, and comment boxes were used to record corresponding notes. In the third stage of analysis, transcripts were imported into the data‐management software NVivo 10, where selections of text were coded to represent instances of a concept.^51^ Codes were reviewed in terms of their relationship to other codes and combined to create more developed themes.^51^ From this analysis, distinctions between the different levels of themes appeared (eg main overarching themes and subthemes within them).

Data extracts were regularly shared with members of the project support group to discuss interpretation of the data and to confirm the emerging themes – improving consistency and reliability of findings.^52^ In order to maintain a progressive, iterative, process of analysis,^53^ data were collected and analysed concurrently, allowing emergent findings to guide, where relevant, the next piece of data collection.

## 3 FINDINGS

Migrant Pakistani participants had a mean age of 27 years old, and mean length of residency in the UK was 7 years. Social characteristics varied, with women from a range of social and economic backgrounds; for example, some had partners in well‐paid professional jobs, whilst others had partners who were manual labourers. Only one of the women was employed. Further demographics of participants are presented in Table 2. All midwife participants were UK‐born and worked in the community. It was decided that further demographic information for this population (such as time in practice or seniority) would act as possible identifiers in such a small sample, so this information is not reported.

**Table 2:** Participant characteristics: migrant Pakistani women

Description	Name	Age	Number of Years in UK	Marital status
Pregnant migrant Pakistani woman	Aleena	32	2	Married
	Eliza	17	13	Married
	Faiza	28	8	Married
	Hana	26	7	Married
	Liyana	30	4	Living with partner
	Nimra	24	3	Married
	Zoya	35	15	Married
Migrant Pakistani language interpreter	Sara	47	10	Married
Mother of Eliza	Rukhsa	56	13	Married

Both participant groups placed high importance on midwife–woman relationships. Not only was this relationship viewed as significant in ensuring the best outcome for women, but it was also suggested that difficult relationships could potentially result in higher risk of adverse pregnancy outcomes, through lack of communication and restricted care. When discussing the factors perceived to influence this client–provider relationship, three key themes were identified within the data: (i) family relationships, (ii) culture and religion and (iii) understanding different health‐care systems. Drawing on relevant data extracts, these themes will now be discussed from the perspectives of both the women and the midwives, noting similarities and differences in viewpoint.

### 3.1 Family relationships

Mothers‐in‐law and domestic partners were seen to play significant roles in women's maternity care by all participants. Whilst women found these family members to be a source of support, midwives, however, perceived this involvement as having a negative impact on midwife–woman relationships. This perception is typified in the following midwife interview extract where mothers‐in‐law are described as “dominating” antenatal clinic appointments, preventing midwives from “really” getting to know women.

Susan (M): You're not quite knowing what the lady herself is thinking. They're inclined to – the mother‐in‐law, if she comes, to sort of dominate the consultation.

Researcher: Do you find that affects your relationship with the client then?

Susan (M): Well it does, really. Because you never really get to **know** them like you do, other ladies. You know…they're keeping them **back**, I think, a little bit really.

The pregnancy and childcare advice given by mothers‐in‐law was also perceived as a potential barrier to midwife–woman relationships, as this advice often conflicted with information provided by midwives.

I mean the ones who have newly come over – all they're hearing is what the mothers‐in‐law or the family tell them…and they're taking that as gospel…And you've got a real battle to say “just because grandma said it doesn't mean to say it's right!” Heather (M)

Women also acknowledged the existence of differing (and often contradictory) pregnancy “knowledge” held by midwives and family elders, reporting confusion and possible tensions resulting from trying to balance sometimes incompatible professional/midwifery and traditional/family ideologies.

I would listen to the midwife. Cos she's obviously the person who's more experienced in that. But then it's tradition….and you kind of respect tradition as well. I don't know – it's a bit difficult. How would you balance it? Eliza (W)

Similarly, the presence of family members, such as domestic partners, at antenatal appointments was also described by midwives as a further barrier to establishing a relationship and getting to know women.

You never get a relationship going how you'd like it to be [if there is another person present]…It's not the same. Even if you've met them once on their own – you kinda get a better idea about who they are. Mary (M)

There was also a high propensity for male partners to speak on behalf of women, even when the woman appeared to have sufficient language skills to communicate directly with the midwife. This behaviour was perceived negatively by midwives, who stereotyped it as an act of male dominance and control.

In their culture the man is the head of the house. So he makes decisions, he does the talking. Gail (M)

Circumstances that result in male partners leading interactions with midwives are illustrated in this fieldnote of booking appointment with a migrant Pakistani woman and her husband.

Woman seems a little nervous – looking to partner for answers when midwife asks questions (despite good English fluency). Partner answers most questions for woman. Partner explains about medical condition of woman. Midwife tries to engage woman by directing questions at her – woman turns to partner and waits for him to answer. Midwife has started to speak to partner more now – directing questions at him instead of woman. Fieldnote 1

Women, however, did not see their partner's behaviour as a barrier to their relationships with midwives, but interpreted it more positively.

I think it's a caring thing. Because they care about their wives and their children. That's why they [speak] for their wives or girlfriends…he speaks for me and he cares about me so I'm happy about it. Liyana (W)

Indeed, some women expressed a preference for family members to speak on their behalf. As typified in the extract below, women were reassured by elders' greater experiences and knowledge, placing a higher priority on the accuracy of the information being communicated than on having one‐to‐one conversations with midwives.

I'd rather have [my mum] talk ‐ she's more experienced with talking to midwives and doctors. And she knows the whole process…I think I'd rather have her talk, than me….If I say something wrong then my mum will be like “no – you say it like this” – that's what I think is important. Eliza (W)

### 3.2 Culture and religion

Although midwives tended to express either positivity or neutrality towards Islam, many expressed concern regarding traditional pregnancy and post‐natal practices rooted in the Muslim culture. Some practices were viewed as unsafe and/or unhygienic; for example dressing newborns with glass/string bracelets, fasting whilst pregnant, shaving the newborn's head or placing honey on the newborn's tongue immediately after birth. Midwives perceived such practices as impacting negatively on the midwife–woman relationship.

Pregnant women shouldn't fast. And I always find that if they are fasting then I'm kind of lecturing them “no – you shouldn't be fasting” and that kind of thing…and they do get a bit funny about it… you can see that they're not happy that I'm saying “no you shouldn't”. Mary (M)

When speaking about practices which they knew to be negatively viewed by health professionals, women tended to discuss the behaviours and attitudes of others. For example, whilst claiming they would personally adhere to the midwife's advice during pregnancy, women suggested that “other” Pakistani women were more likely to agree with the midwife superficially, whilst continuing practices out of the health professional's sight.

Whatever [midwife] say, [Pakistani women] won't follow you. They will say “ok yes we will do” in front of you…but when they go back home they won't follow you! They will follow whatever the elders say – they will follow that! Hana (W)

Continuation of practices against advice was also cited by midwives as a barrier to good midwife–woman relationships. Midwives expressed anxieties about balancing professional accountability with providing choice, personalized services and safe care to women and babies.

Even without personal experience of women's traditional practices or views leading to adverse outcomes, midwives continued to negatively stereotype migrant Pakistani women. For example, midwives described how they thought Muslim women were more likely to accept a stillbirth as “God's will” and were therefore less likely to seek medical help for pregnancy concerns. Women, on the other hand, suggested that this belief provided them with a way of coping with negative outcomes, but that it did not negate the need for antenatal care.

Interestingly, data suggested a bidirectional effect between midwives' views on traditional pregnancy practices and the establishment of the midwife–woman relationship: when a positive midwife–woman relationship was established, midwives tended to view traditional practices such as head shaving more positively. However, the extract below demonstrates that if a positive relationship was not yet established, midwives could harshly judge some of the women's decision making.

If you haven't built up a relationship with somebody during pregnancy…you tend to actually be very hard on some of the decisions they make, and I think we need to be honest about it. Gail (M)

### 3.3 Understanding different health‐care systems

Whilst midwives spoke about UK maternity care as the “gold standard,” some women stated a preference for the Pakistani maternity care system, particularly citing the lack of woman‐initiated contact at the beginning of pregnancy as a potential problem for the on‐going relationship with midwives.

We can't contact our midwife, or visit them frequently…. [Pakistani women] would be more happy if they had care or attention in the beginning. Because that period is more sensitive and more need to care…I think it will make good relationship between you and your midwife. Hana (W)

Although the women in my study appeared to trust the quality of the maternity care in South Wales, many admitted to being unsure about the qualifications and professional responsibilities of UK midwives. This lack of clarity continued even after women had attended antenatal appointments.

I don't have any idea about midwife – I mean – what they do, how much qualified they are. Seriously – at this stage I really don't know. Hana (W)

The biggest source of tension for midwives was caused by migrant Pakistani women (in addition to other non‐British women) arriving late to antenatal appointments or missing these appointments altogether. Midwives' frustrations stemmed not only from non‐attendance, but also from the perceived reasons behind this non‐attendance. For example, midwives stereotyped most non‐British women as being unconcerned by the time pressures resulting from late, or non‐arrival for an appointment.

That's one of the banes of my clinic. I give them appointments and they just turn up when they like.. [They] know they're late and they've missed their time – but they still do it! Mary (M)

My own experiences over the course of data collection supported this perception, as demonstrated in the fieldnote extract below.

Woman is late by 40 minutes – midwife now pressured to do an hour booking in 20mins. Midwife obviously a bit stressed about this but puts on cool, calm face when woman enters. Woman doesn't seem concerned that she is late – no apology. Fieldnote 2

In contrast to the views of midwives, women suggested that reasons for non‐ or late attendance at antenatal appointments included misunderstanding and poor knowledge of UK health‐care system norms. For example, women spoke of busy domestic lives, limited transport options and confusion regarding pre‐booked appointments.

[In Pakistan] they just go there, straight away. Take a number and sit. And when they call them – they go and tell the doctor what's going on. And that's why people don't know about appointments [here], you know, to make them. Sara (Interpreter)

Additionally, some midwives described situations where women viewed antenatal care as unnecessary, as they were not unwell. Others suggested that migrant women only attended antenatal care when they wanted signatures and referral letters for other agencies, rather than a desire for what midwives considered as actual *care*. From the midwives' perspective, there seemed to be a lack of reciprocity in these interactions; midwives attempted to provide holistic care to women, whilst women rejected this approach in favour of a more administrative or task focussed role of the midwife. Repeated requests from women for this type of support seemed to cause tensions in relationships.

What I dislike is people who come in and they've got a list of demands. “You need to write me a letter for housing. You need to do this – you need to do that” That's all they want! Care isn't always a priority for them…they'll only come when they want something. Gail (M)

### 3.4 Connecting the themes

Despite commonalities in the maternity care issues identified by women and midwives (family involvement, culture and religion, differing health‐care systems), differences were observed in the way midwives and women prioritized these topics in terms of their influence on the midwife–woman relationship. For example, midwives most commonly attributed poor relationships with migrant women firstly to misunderstanding of, or lack of adaptation to the health‐care system generally and secondly to misunderstanding the nature of the midwife–woman relationship specifically (eg that family members were not expected to contribute when midwives met with women). In contrast, women tended to centre their accounts around the importance of family involvement in their maternity care and their expectations that services adapt to their needs.

Two interweaving themes were also identified from the data, which linked the main themes. The first of these was labelled “authoritative knowledge” and was used to refer to instances where competing sources of knowledge seemed to disrupt midwife–woman relationships (eg the incompatibility of midwives and mothers‐in‐law's pregnancy knowledge). The second of these themes, “communication of information,” was identified when important differences between midwives and women in terms of the perceived purpose of communication were noted. For example, midwives placed importance on social aspects of communication in order to build a close relationship, such as small talk, humour and women's hobbies and interests, whilst women prioritized the factual correctness of the information being communicated, rather than focusing on small talk.

## 4 DISCUSSION

Findings from this research build on previous literature which suggests that midwife–woman relationships are important for women's experiences of maternity care, pregnancy outcomes and staff satisfaction.^17^, ^29^, ^54^ However, this study provides new knowledge in this field by identifying factors which influence the creation and maintenance of this relationship between UK‐born midwives and migrant Pakistani women in the UK. Furthermore, these novel findings suggest substantial differences in the way midwives and women perceive the influence of these factors on experiences of maternity care and midwife–woman relationships, suggesting that divergent expectations must be addressed and managed if positive relationships are to be established and maintained.

Both participant groups acknowledged that the involvement of women's family members in maternity care had the potential to influence midwife–woman relationships, especially when it came to competing advice about pregnancy and post‐natal practices. Indeed, participants identified that midwives often held differing (and sometimes incompatible) pregnancy “knowledge” to family elders and that this could cause tension throughout women's maternity care. The existing literature hints at similar tensions between health‐care practitioners and mothers/mothers‐in‐law of migrant women, where staff report not wanting to “tackle the grandmother” [Ref.^55^, p.131]. More specifically, competition between different sources of knowledge is a long‐standing issue in birth settings^56^, ^57^, ^58^, ^59^ and is characterized as a struggle for “authoritative knowledge”^60^ where: “for any particular domain several knowledge systems exist, some of which, by consensus, come to carry more weight” [Ref.^60^, p.56]. Although similar tensions over legitimacy of knowledge are reported between UK midwives and UK grandmothers,^61^ such disagreements are arguably more likely between members of different cultures, where traditional cultural practices are likely to diverge. Interestingly, findings from this study suggested that women struggled with decisions regarding competing knowledge sources and expressed a desire to follow both the advice of midwives and elders. We would suggest, therefore, that building good relationships with (and educating) grandmothers during antenatal contact may negate or reduce issues of competing knowledge and tension in midwife–woman relationships for some migrant women.

Muslim culture and religion were also seen to influence midwife–woman relationships through midwives' perceptions of the role of faith in determining pregnancy outcomes. Midwives expressed concerns that such beliefs downplayed the role of antenatal care and voiced anxieties regarding their professional and legal accountability for decisions made by women in terms of their engagement in care. Previous research has noted similar concerns from midwives who expressed frustration regarding first‐generation migrant Somali women's reliance on “the work of Allah” [Ref.^33^, p.14] in their pregnancies. It is possible that such anxieties stem from the concept of “informed choice” in midwifery practice,^61^ whereby midwives are tasked with providing all of the appropriate information to women and then responsibility is placed on both parties to influence care and outcomes.^62^, ^63^ Whilst accountability is arguably applicable in all cases where care decisions are shared with women themselves, midwives may feel more concern about decisions regarding practices they are unfamiliar with, compared to more familiar decisions made by UK‐born women. This may explain research findings which suggest that midwives describe their job and relationships with women as more demanding, difficult and stressful when working with migrant women.^33^, ^64^ Consequently, these findings suggest that more needs to be done to address midwives' concerns around the safety of unfamiliar practices, for example having standardized evidence‐based information on such practices available to give to women. Such actions may allow midwife–woman tensions to be reduced and positive relationships to be maintained.

The lack of lone contact with migrant women was also highlighted as a concern by midwives, who suggested that the presence of male partners not only negated the possibility of conducting routine enquiry but also prevented the establishment of a good midwife–woman relationship. Minimum standards for midwifery in Wales posit that midwives should ensure lone contact with all women at least once in their pregnancy and that women should be alone when asked about domestic abuse (a routine enquiry which should occur at least once during antenatal care).^65^ Such standards are therefore likely to frame the way in which midwives in Wales view partner involvement and could account for the anxieties expressed by midwives in this research. Despite the priority given to this issue by midwives, women seemed unaware that their partner's involvement in their antenatal care might affect their relationships with midwives and instead framed the partner's involvement as positive and caring. It is our view, therefore, that addressing these expectations of lone midwife–woman contact with both parties at an early stage of maternity care engagement could go some way to reducing the potential for tension or misunderstanding regarding partner involvement in care and therefore improve relationships between midwives and migrant women.

Expectations of the UK maternity care system also differed between midwives and women, and this was especially apparent when discussing women's navigation of maternity care. Indeed, late or non‐attendance at antenatal appointments was seen by midwives to be one of the biggest influences on their relationships with women. Similar tensions regarding ethnic minority and migrant women's navigation of maternity care systems are found in the existing literature. For example, during interviews with Lyons et al,^66^ maternity care providers reported that when minority ethnic women arrived late or missed antenatal appointments, they were breaking the “unwritten” rules of behaviour of the hospital. Lack of conformity to these behavioural norms was seen to cause negative responses from staff,^66^ a finding which is reflected by our own data. However, findings from the current study differ slightly from previous research in terms of the explanations given for non‐attendance by the women themselves; whilst previous literature from the UK cites language barriers as the main challenge to migrant women accessing health care,^67^, ^68^ women in our study suggested that late or non‐attendance was most commonly the result of difficulty navigating the UK health‐care system. This finding therefore offers new potential ways to align expectations around migrant women's navigation of health‐care systems, and improve midwife–woman relationships for this client group. For example, more could be done to ensure women's understanding of appointment systems by educating women on the UK maternity system at the point of migration or initial contact with UK health‐care services.

Differences also existed between midwives and women in terms of expectations regarding the function of antenatal care and the professional role held by UK midwives. Indeed, the UK “partnership approach”^69^ to care, which emphasizes equal division of labour and responsibility between health‐care provider and woman,^70^, ^71^ was mostly rejected by women in the current study, in favour of advice from family members. Mirroring findings from previous research,^61^, ^71^ midwives in our research cited women's rejection of this partnership way of working as a contributing factor to complex and tense midwife–woman interactions. Similarly, women's own unmet expectations regarding antenatal care were also seen to inhibit the creation of a good midwife–woman relationship. These findings are supported by the existing literature, which suggests that migrant women who have limited knowledge of UK maternity care often expect care similar to that provided in their country of origin,^64^ and can therefore become dissatisfied with care which differs from these expectations.^64^

Overall, midwife–woman relationships appeared to be influenced by divergent expectations and priorities placed on aspects of maternity care, such as family involvement, culture and religion, and navigating health‐care systems. However, it is important to note the existence of individual differences in terms of expectations and priorities and to acknowledge that some pairings of midwives and women will converge more closely on these factors than others. Therefore, it is our recommendation that expectations of UK maternity care are addressed and managed not only at a global level (ie all midwives and women) but also on an individual level (ie exploring and managing expectations for each individual pairing of midwife and woman).

### 4.1 Strengths and limitations

The methodological approach taken by this research provides one of the most in‐depth ethnographic studies of this topic area since Bowler's work investigating South Asian women's maternity experiences in 1993.^32^ As such, the current study delivers an updated insight into the lived experiences of, and relationships between, midwives and pregnant migrant women in the UK. Furthermore, this study expands knowledge in the under‐researched area of maternity care experiences in the Welsh context, where maternity policy and health inequality policies differ to those in England.^72^, ^73^, ^74^

As with all studies, results should be interpreted in the context of limitations. This research was conducted in a single health region, with recruitment of participants linked to the services provided in this region. It is therefore possible that the findings of this research may not be generalizable to other geographical areas, especially those outside South Wales. However, in keeping with an ethnographic approach, the richness of data was prioritized over generalizability, providing a nuanced, empirically rich, holistic account of two specific “cultures,” that takes into account contextual factors such as local characteristics.^75^, ^76^, ^77^

## 5 CONCLUSIONS

Ethnic and migrant inequality in pregnancy outcomes is an increasingly important area of study as achieving better outcomes for migrant and ethnic minority communities is of global concern. In the UK specifically, growing numbers of migrant women are accessing maternity care each year,^3^ and their relative risk of maternal death appears to be increasing.^13^ Better understanding of the relationship between midwives and migrant woman at this key time in a woman's life may contribute to addressing some of these challenges.

Findings from this study provide new theoretical insights into the complex factors contributing to the health‐care expectations of pregnant migrant Pakistani women in the UK and the ways in which these expectations influence midwife–woman relationships for this population. The differences seen between midwives and women in the perceived importance of these factors suggest that, in order to understand how midwife–woman relationships are created and maintained, more needs to be done to recognize and manage differing expectations of maternity care.

## CONFLICT OF INTEREST

Billie Hunter is the Royal College of Midwives (RCM) Professor of Midwifery, and her post is partly funded by the RCM. There are no other conflicts of interest to declare.

## References

[1] Office for National Statistics , Statistical bulletin: Migration Statistics Quarterly Report: February 2016. 2016: http://www.ons.gov.uk/peoplepopulationandcommunity/populationandmigration/internationalmigration/bulletins/migrationstatisticsquarterlyreport/february2016. Accessed August 5, 2016.

[2] Rienzo C , Vargas‐Silva C . Migrants in the UK: An Overview. Migration Observatory Briefing. 2016, COMPAS, University of Oxford.: http://www.migrationobservatory.ox.ac.uk/briefings/migrants-uk-overview. Accessed August 7, 2016.

[3] Office for National Statistics . Statistical bulletin: Parents' country of birth, England and Wales: 2015 https://www.ons.gov.uk/peoplepopulationandcommunity/birthsdeathsandmarriages/livebirths/bulletins/parentscountryofbirthenglandandwales/2015 2016]. Accessed February 17, 2017.

[4] Redshaw M , Rowe R , Hockley C , Brocklehurst P . Recorded Delivery: A National Survey of Women's Experience of Maternity Care. Oxford: National Perinatal Epidemiology Unit (NPEU); 2007.

[5] Richens Y . Exploring the Experiences of Women of Pakistani Origin of UK Maternity Services. London: Department of Health; 2003.

[6] Bowes A , Domokos TM . Your dignity is hung up at the door: Pakistani and White Women's experiences of childbirth, In: Earle S, Letherby G, eds. Gender, Identity & Reproduction: Social Perspectives. London: Palgrave Macmillan UK; 2003:87‐102.

[7] Singh D , Newburn M . Women's Experiences of Postnatal Care. London: National Childbirth Trust; 2000.

[8] Redshaw M , Heikkila K . Ethnic differences in women's worries about labour and birth. Ethn Health. 2011;16:213‐223. 21500115 10.1080/13557858.2011.561302

[9] Nair M , et al. Factors associated with maternal death from direct pregnancy complications: a UK national case–control study. BJOG. 2015;122:653‐662. 25573167 10.1111/1471-0528.13279 PMC4674982

[10] Essex HN , Green J , Baston H , Pickett KE . Which women are at an increased risk of a caesarean section or an instrumental vaginal birth in the UK: an exploration within the Millennium Cohort Study. BJOG. 2013;120:732‐743. 23510385 10.1111/1471-0528.12177

[11] Raleigh VS , Hussey D , Seccombe I , Hallt K . Ethnic and social inequalities in women's experience of maternity care in England: results of a national survey. J R Soc Med. 2010;103:188‐198. 20436027 10.1258/jrsm.2010.090460 PMC2862068

[12] Knight M , Tuffnell D , Kenyon S , Shakespeare J , Gray R , Kurinczuk J . Saving Lives, Improving Mothers' Care ‐ Surveillance of maternal deaths in the UK 2011‐13 and lessons learned to inform maternity care from the UK and Ireland Confidential Enquiries into Maternal Deaths and Morbidity 2009‐13. 2015.

[13] Knight M , Nair M , Tuffnell D , Kenyon S , Shakespeare J , Brocklehurst P , Kurinczuk J . Saving lives, improving mothers' care. Surveillance of maternal deaths in the UK 2012‐2014 and lessons learned to inform maternity care from the UK and Ireland Confidential Enquiries into Maternal Deaths and Morbidity 2009‐2014. 2016, MBRRACE‐UK: Oxford.

[14] Draper E , Kurinczuk J , Kenyon S . MBRRACE‐UK Perinatal Confidential Enquiry: Term, singleton, normally formed, antepartum stillbirth. 2015, MBRRACE‐UK: Leicester.

[15] Manktelow B , Smith LK , Prunet C , et al. Perinatal Mortality Surveillance Report. UK Perinatal Deaths for births from January to December 2013, MBRRACE‐UK, Ed. 2015: Leicester.

[16] Hayes I , Enohumah K , McCaul C . Care of the migrant obstetric population. Int J Obstet Anesth. 2011;20:321‐329. 21840201 10.1016/j.ijoa.2011.06.008

[17] Puthussery S , Twamley K , Macfarlane A , Harding S , Baron M . ‘You need that loving tender care’: maternity care experiences and expectations of ethnic minority women born in the United Kingdom. J Health Serv Res Policy. 2010;15:156‐162. 20466754 10.1258/jhsrp.2009.009067

[18] Puthussery S , Twamley K , Harding S , Mirsky J , Baron M , Macfarlane A . ‘They're more like ordinary stroppy British women’: attitudes and expectations of maternity care professionals to UK‐born ethnic minority women. J Health Serv Res Policy. 2008;13:195‐201. 18806176 10.1258/jhsrp.2008.007153

[19] Zwart JJ , Jonkers MD , Richters A , Ory F , Bloemenkamp KW , Duvekot JJ , van Roosmalen J . Ethnic disparity in severe acute maternal morbidity: a nationwide cohort study in the Netherlands. Eur J Public Health. 2011;21:229‐234. 20522516 10.1093/eurpub/ckq046

[20] Naran NH , Chetty N , Crowther NJ . The influence of metabolic syndrome components on plasma PAI‐1 concentrations is modified by the PAI‐1 4G/5G genotype and ethnicity. Atherosclerosis. 2008;196:155‐163. 17467713 10.1016/j.atherosclerosis.2007.03.024

[21] Giger J , Davidhizar R . Transcultural Nursing: Assessment and Intervention. 3rd edn. St Louis, MO: C. V. Mosby; 1999,

[22] Office for National Statistics , Immigration Patterns of Non‐UK Born Populations in England and Wales in 2011. 2013.

[23] Jonkers M , Richters A , Zwart J , Ory F , van Roosmalen J . Severe maternal morbidity among immigrant women in the Netherlands: patients' perspectives. Reprod Health Matters. 2011;19:144‐153. 21555095 10.1016/S0968-8080(11)37556-8

[24] Mustillo S , Krieger N , Gunderson EP , Sidney S , McCreath H , Kiefe CI . Self‐reported experiences of racial discrimination and Black‐White differences in preterm and low‐birthweight deliveries: the CARDIA Study. Am J Public Health. 2004;94:2125‐2131. 15569964 10.2105/ajph.94.12.2125 PMC1448602

[25] Knight M , Kurinczuk JJ , Spark P , Brocklehurst P . Inequalities in maternal health: national cohort study of ethnic variation in severe maternal morbidities. BMJ. 2009;338:b542. 19261591 10.1136/bmj.b542 PMC2654771

[26] Goodwin L , Hunter B , Jones A . Immigration and Continuing Inequalities in Maternity Outcomes: Time to Reexplore the Client– Provider Relationship? Int J Childbirth. 2015;5:12‐19.

[27] Lewis G . The Confidential Enquiry into Maternal and Child Health (CEMACH). Saving Mothers' Lives: reviewing maternal deaths to make motherhood safer ‐ 2003‐2005. The Seventh Report on Confidential Enquiries into Maternal Deaths in the United Kingdom. 2007, CEMACH: London.

[28] Lynch N . Women's Experiences of Maternity Services in Birmingham East and North and Solihull. A Qualitative Study. 2011, Involvement Innovation Ltd.

[29] Cantwell R , et al. Saving Mothers' Lives: Reviewing maternal deaths to make motherhood safer: 2006‐2008. The Eighth Report of the Confidential Enquiries into Maternal Deaths in the United Kingdom. BJOG. 2011;118(Suppl 1):1‐203. 10.1111/j.1471-0528.2010.02847.x 21356004

[30] Hunter B . The importance of reciprocity in relationships between community‐based midwives and mothers. Midwifery. 2006;22:308‐322. 16616398 10.1016/j.midw.2005.11.002

[31] Hunter B , et al. Relationships: the hidden threads in the tapestry of maternity care. Midwifery. 2008;24:132‐137. 18378051 10.1016/j.midw.2008.02.003

[32] Bowler I . ‘They're not the same as us’: midwives' stereotypes of South Asian descent maternity patients. Sociol Health Illn. 1993;15:157‐178.

[33] Essén B , Binder P , Johnsdotter S . An anthropological analysis of the perspectives of Somali women in the West and their obstetric care providers on caesarean birth. J Psychosom Obstet Gynaecol. 2011;32:10‐18. 21291343 10.3109/0167482X.2010.547966 PMC3055712

[34] Office for National Statistics , Statistical bulletin: 2011 Census: Detailed Characteristics for England and Wales, March 2011. 2013: http://www.ons.gov.uk/peoplepopulationandcommunity/populationandmigration/populationestimates/bulletins/2011census/2013-05-16. Accessed 16 May, 2013.

[35] Hammersley M , Atkinson P . Ethnography: Principles in Practice, 2nd edn. London: Routledge; 1995.

[36] Holloway I , Wheeler S . Qualitative Research for Nurses. Oxford: Blackwell Science; 1996.

[37] O'Reilly K . Ethnographic Methods. London: Routledge; 2012.

[38] Higginbottom G , Pillay JJ , Boadu NY . Guidance on Performing Focused Ethnographies with an Emphasis on Healthcare Research. Qual Rep. 2013;18:1‐6.

[39] Cruz EV , Higginbottom G . The use of focused ethnography in nursing research. Nurse Res. 2013;20:36‐43. 23520711 10.7748/nr2013.03.20.4.36.e305

[40] Roper JM , Shapira J . Ethnography in Nursing Research. Vol. 1. Thousand Oaks, California: Sage; 2000.

[41] Nightingale R , Sinha MD , Swallow V . Using focused ethnography in paediatric settings to explore professionals' and parents' attitudes towards expertise in managing chronic kidney disease stage 3–5. BMC Health Serv Res. 2014;14:403. 25234741 10.1186/1472-6963-14-403 PMC4176584

[42] Liamputtong P , Ezzy D . Qualitative Research Methods (2nd edn). Melbourne, AU: Oxford University Press; 2005.

[43] Cross RM . Exploring attitudes: the case for Q methodology. Health Educ Res. 2005;20:206‐213. 15385430 10.1093/her/cyg121

[44] Atkinson P , et al. Handbook of Ethnography. London (UK): SAGE Publications; 2001.

[45] StatsWales , Medical and dental staff by specialty and year. 2015: https://statswales.gov.wales/Catalogue/Health-and-Social-Care/NHS-Staff/Medical-and-Dental-Staff/hospitalmedicalanddentalstaff-by-specialty-year. Accessed August 19, 2016.

[46] Jivraj S . Geographies of Diversity in Cardiff, in Local Dynamics of Diversity: evidence from the 2011 Census. Manchester (UK): Centre on Dynamics of Ethnicity, The University of Manchester; 2013.

[47] Strauss A , Corbin J . Basics of Qualitative Research: Grounded Theory Procedures and Techniques. Newbury Park, CA: Sage publications; 1990.

[48] Kuzel AJ . Sampling in qualitative inquiry In: Crabtree B, Miller W, eds. Doing Qualitative Research: Research Methods for Primary Care. London: SAGE Publications, Inc; 1992:31‐44.

[49] Ross‐Davie MC , Cheyne H , Niven C . Measuring the quality and quantity of professional intrapartum support: testing a computerised systematic observation tool in the clinical setting. BMC Pregnancy Childbirth. 2013;13:1‐12. 23945049 10.1186/1471-2393-13-163 PMC3751507

[50] Braun V , Clarke V . Using thematic analysis in psychology. Qual Res Psychol. 2006;3:77‐101.

[51] Bazeley P , Jackson K . Qualitative Data Analysis with NVivo. Thousand Oaks, California: Sage Publications Limited; 2013.

[52] Pope C , Ziebland S , Mays N . Qualitative research in health care. Analysing qualitative data. BMJ. 2000;320:114‐116. 10625273 10.1136/bmj.320.7227.114 PMC1117368

[53] Srivastava P , Hopwood N . A Practical Iterative Framework for Qualitative Data Analysis. Int J Qual Methods. 2009;8:76‐84.

[54] Tinkler A , Quinney D . Team midwifery: the influence of the midwife‐woman relationship on women's experiences and perceptions of maternity care. J Adv Nurs. 1998;28:30‐35. 9687127 10.1046/j.1365-2648.1998.00769.x

[55] McFadden A , Renfrew MJ , Atkin K . Does cultural context make a difference to women's experiences of maternity care? A qualitative study comparing the perspectives of breast‐feeding women of Bangladeshi origin and health practitioners. Health Expect. 2012;16:124‐135. 10.1111/j.1369-7625.2012.00770.x PMC5060684 22429489

[56] Irwin S , Jordan B . Knowledge, Practice, and Power: Court‐Ordered Cesarean Sections. Med Anthropol Q. 1987;1:319‐334. 11659066 10.1525/maq.1987.1.3.02a00060

[57] Jordan B . The hut and the hospital: information, power, and symbolism in the artifacts of birth. Birth. 1987;14:36‐40. 3646889 10.1111/j.1523-536x.1987.tb01446.x

[58] Hunt LM , Jordan B , Irwin S . Views of what's wrong: diagnosis and patients' concepts of illness. Soc Sci Med. 1989;28:945‐956. 2711229 10.1016/0277-9536(89)90324-9

[59] Hunt LM , Jordan B , Irwin S , Browner CH . Compliance and the patient's perspective: controlling symptoms in everyday life. Cult Med Psychiatry. 1989;13:315‐334. 2776467 10.1007/BF00054341

[60] Jordan B . Authoritative knowledge and its construction. Childbirth and authoritative knowledge: Cross‐cultural perspectives, 1997:55‐79.

[61] Sanders J , Hunter B , Warren L . A wall of information? Exploring the public health component of maternity care in England. Midwifery. 2015;34:253‐260. 26608787 10.1016/j.midw.2015.10.013

[62] Department of Health , Maternity Matters: Choice, access and continuity of care in a safe service. 2007: London.

[63] Cross‐Sudworth F , Williams A , Herron‐Marx S . Maternity services in multi‐cultural Britain: using Q methodology to explore the views of first‐ and second‐generation women of Pakistani origin. Midwifery. 2011;27:458‐468. 21036439 10.1016/j.midw.2010.03.001

[64] Aquino MRJV , Edge D , Smith DM . Pregnancy as an ideal time for intervention to address the complex needs of black and minority ethnic women: Views of British midwives. Midwifery. 2015;31:373‐379. 25483209 10.1016/j.midw.2014.11.006

[65] All Wales Midwifery & Health Visitors Domestic Abuse Networking Group , All Wales Pathway. Antenatal Routine Enquiry into Domestic Abuse: Minimum Standards. 2006, Welsh Assembly Government.

[66] Lyons SM , et al. Cultural diversity in the Dublin maternity services: the experiences of maternity service providers when caring for ethnic minority women. Ethn Health. 2008;13:261‐276. 18568976 10.1080/13557850801903020

[67] Threadgold T , et al. Constructing community in South‐East Wales. 2007: http://www.cardiff.ac.uk/jomec/resources/ConstructingCommunity.pdf. Accessed August 25, 2016.

[68] Harper Bulman K , McCourt C . Somali refugee women's experiences of maternity care in west London: A case study. Crit Public Health, 2002;12:365‐380.

[69] Royal College of Midwives , High Quality Midwifery Care. 2014: https://www.rcm.org.uk/sites/default/files/High%20Quality%20Midwifery%20Care%20Final.pdf. Accessed August 25, 2016.

[70] Bekker HL . Genetic screening: facilitating informed choices. eLS, 2003.

[71] Boyle S . Women's views on partnership working with midwives during pregnancy and childbirth. 2013, University of Hertfordshire. 10.1016/j.midw.2015.09.001 26597110

[72] Public Health England , National Conversation on Health Inequalities. 2015: https://www.gov.uk/government/collections/national-conversation-on-health-inequalities. Accessed September 15, 2016.

[73] Welsh Government , A Strategic Vision for Maternity Services in Wales. 2011: http://www.wales.nhs.uk/documents/A%20Strategic%20Vision%20for%20Maternity%20Services%20in%20Wales%20-%20September%202011.pdf. Accessed September 14, 2016.

[74] National Maternity Review, Better Births. Improving outcomes of maternity services in England. A Five Year Forward View for maternity care. 2016.

[75] Geertz C . The Interpretation of Cultures: Selected Essays. Vol. 5019. London (UK): Basic books; 1973.

[76] Marcus GE . Ethnography Through Thick and Thin. Princeton, NJ: Princeton University Press; 1998.

[77] Falzon M‐A . Multi‐Sited Ethnography: Theory, Praxis and Locality in Contemporary Research. London (UK): Routledge; 2016.

